# Comparison of first-line treatment with CHOP versus ICED in patients with peripheral T-cell lymphoma eligible for upfront autologous stem cell transplantation

**DOI:** 10.3389/fonc.2023.1230629

**Published:** 2023-08-22

**Authors:** Seok Jin Kim, Jae-Cheol Jo, Dok Hyun Yoon, Deok-Hwan Yang, Sang Eun Yoon, Gyeong-Won Lee, Jee Hyun Kong, Yong Park, Ka-Won Kang, Ho-Sup Lee, Sung Yong Oh, Ho-Jin Shin, Won Sik Lee, Yoon Seok Choi, Seong Hyun Jeong, Min Kyoung Kim, Hye Jin Kang, Jun Ho Yi, Sung-Nam Lim, Ho-Young Yhim, Young Rok Do, Hwan Jung Yun, Hyeon-Seok Eom, Mark Hong Lee, Cheolwon Suh, Won Seog Kim

**Affiliations:** ^1^ Division of Hematology-Oncology, Department of Medicine, Samsung Medical Center, Sungkyunkwan University School of Medicine, Seoul, Republic of Korea; ^2^ Department of Health Sciences and Technology, Samsung Advanced Institute for Health Sciences and Technology, Sungkyunkwan University School of Medicine, Seoul, Republic of Korea; ^3^ Department of Hematology and Oncology, Ulsan University Hospital, University of Ulsan College of Medicine, Ulsan, Republic of Korea; ^4^ Department of Oncology, University of Ulsan College of Medicine, Asan Medical Center, Seoul, Republic of Korea; ^5^ Department of Internal Medicine, Chonnam National University Hwasun Hospital, Chonnam National University Medical School, Gwangju, Republic of Korea; ^6^ Division of Hematology and Oncology, Department of Internal Medicine, Gyeongsang National University Hospital, Gyeongsang National University College of Medicine, Changwon, Republic of Korea; ^7^ Department of Hematology-oncology, Division of Internal Medicine, Wonju Severance Christian Hospital, Yonsei University Wonju College of Medicine, Wonju, Republic of Korea; ^8^ Cancer of Evidence Based Medicine, Institute of Convergence Science, Yonsei University, Seoul, Republic of Korea; ^9^ Department of Internal Medicine, Korea University College of Medicine, Korea University Anam Hospital, Seoul, Republic of Korea; ^10^ Department of Internal Medicine, Kosin University Gospel Hospital, Busan, Republic of Korea; ^11^ Department of Internal Medicine, Dong-A University Medical Center, Busan, Republic of Korea; ^12^ Department of Internal Medicine, Pusan National University Hospital, Busan, Republic of Korea; ^13^ Department of Internal Medicine, Inje University Busan Paik Hospital, Busan, Republic of Korea; ^14^ Division of Hematology-Oncology, Department of Internal Medicine, Ajou University School of Medicine, Suwon, Republic of Korea; ^15^ Department of Internal Medicine, Yeungnam University College of Medicine, Daegu, Republic of Korea; ^16^ Department of Internal Medicine, Korea Cancer Center Hospital, Korea Institute of Radiological and Medical Sciences, Seoul, Republic of Korea; ^17^ Department Internal Medicine, Chung-Ang University Hospital, Seoul, Republic of Korea; ^18^ Department of Internal Medicine, Haeundae Baek Hospital, Busan, Republic of Korea; ^19^ Department of Internal Medicine, Jeonbuk National University Medical School, Research Institute of Clinical Medicine of Jeonbuk National University-Biomedical Research Institute of Jeonbuk National University Hospital, Jeonju, Republic of Korea; ^20^ Department of Internal Medicine, Dongsan Medical Center, Daegu, Republic of Korea; ^21^ Department of Internal Medicine, Chungnam National University Hospital, Daejeon, Republic of Korea; ^22^ Hematology-Oncology Clinic, National Cancer Center, Go-Yang, Republic of Korea; ^23^ Division of Hematology-Oncology, Department of Internal Medicine, Konkuk University Medical Center, Seoul, Republic of Korea

**Keywords:** T-cell, lymphoma, chemotherapy, autologous stem cell transplantation, progression-free survival

## Abstract

**Introduction:**

Upfront autologous stem cell transplantation (ASCT) has been recommended for patients who are newly diagnosed with peripheral T-cell lymphoma (PTCL), and CHOP (cyclophosphamide, doxorubicin, vincristine, and prednisone), an anthracycline-based chemotherapy has been the frontline chemotherapy for PTCL. However, it is not clear whether anthracycline-based chemotherapies such as CHOP could be standard induction therapy for PTCL.

**Methods:**

We conducted a randomized phase II study to compare CHOP with fractionated ifosfamide, carboplatin, etoposide, and dexamethasone (ICED) for patients eligible for ASCT. The primary endpoint was progression-free survival (PFS) and secondary endpoints included objective response rate, overall survival (OS), and safety profiles.

**Results:**

Patients were randomized into either CHOP (n = 69) or ICED (n = 66), and the characteristics of both arms were not different. PTCL-not otherwise specified (NOS, n = 60) and angioimmunoblastic T-cell lymphoma (AITL, n = 53) were dominant. The objective response rate was not different between CHOP (59.4%) and ICED (56.1%), and the 3-year PFS was not different between CHOP (36.7%) and ICED (33.1%). In AITL patients, CHOP was favored over ICED whereas ICED was associated with more cytopenia and reduced dose intensity. Patients who received upfront ASCT after achieving complete response to CHOP or ICED showed 80% of 3-year OS.

**Discussion:**

In summary, our study showed no therapeutic difference between CHOP and ICED in terms of response and PFS. Thus, CHOP might remain the reference regimen especially for AITL based on its better outcome in AITL, and upfront ASCT could be recommended as a consolidation of complete response in patients with PTCL.

## Introduction

Peripheral T-cell lymphomas (PTCLs) are a heterogeneous group of non-Hodgkin lymphomas including PTCL-not otherwise specified (PTCL-NOS), angioimmunoblastic T-cell lymphoma (AITL), and anaplastic lymphoma kinase (ALK)-positive or -negative anaplastic large-cell lymphoma (ALCL) (1, 2). Their prognoses are still poor because of frequent relapses and the absence of optimal standard therapy for previously untreated patients with PTCLs (3, 4). Accordingly, clinical trials are preferred for stage I–IV, treatment-naive patients with PTCL-NOS, AITL, and ALK-negative ALCL. Cyclophosphamide, doxorubicin, vincristine, and prednisone (CHOP) is adopted as the reference regimen because it is widely used in clinical practice for most patients, and upfront autologous stem cell transplantation (ASCT) is considered as consolidation (5, 6). While previous phase II trials have aimed to improve the efficacy of CHOP by combining it with drugs with a different mode of action such as bortezomib, everolimus, or bevacizumab as frontline therapy, those studies have failed to show an improved outcome in patients with PTCLs (7–9).

Those unsatisfactory outcomes of CHOP might be associated with resistance to anthracyclines, raising the possibility that non-anthracycline chemotherapy regimens might be a treatment option for patients with PTCLs such as natural killer/T-cell lymphoma (10). In a large cohort study of the German High-Grade Non-Hodgkin Lymphoma Study Group, the addition of etoposide to CHOP (CHOEP) improved three-year event-free survival, supporting the role of etoposide in the treatment of PTCLs (11). Likewise, previous retrospective studies have reported that addition of ifosfamide, carboplatin, and etoposide (ICE) or ifosfamide, vincristine, and etoposide (IVE) to CHOP produced favorable outcomes in treatment-naive patients with PTCLs (12, 13). However, the direct comparison between anthracycline and non-anthracycline regimens such as CHOP versus ICE or ICE-like regimens has never been prospectively studied in patients newly diagnosed with PTCLs. Furthermore, there are few prospective data regarding the performance of upfront ASCT in patients with PTCLs. The Nordic Lymphoma Group (NLG) reported the encouraging outcomes of upfront ASCT in a large prospective study with treatment-naive patients with PTCL (14). However, all patients received an induction therapy of six cycles of biweekly CHOP plus etoposide. Therefore, we designed a randomized phase II study comparing CHOP with a non-anthracycline regimen consisting of ifosfamide, carboplatin, etoposide, and dexamethasone (ICED), a regimen adjusted from the ICE regimen by dividing the infusion of ifosfamide into 3 days and adding high-dose dexamethasone, as previous studies reported fractionated ICED might be a reasonable replacement for the classic ICE regimen (15, 16). In 2015, the Consortium for Improving Survival of Lymphoma (CISL) started a randomized phase II study comparing ICED with CHOP as an induction treatment and upfront ASCT in transplant-eligible patients with newly diagnosed, treatment-naive PTCLs. In this study, we present the final analysis of our phase II randomized study.

## Subjects and methods

### Study design and participants

This study was a phase II, multicenter, open-label randomized trial at 21 hospitals that belonged to the CISL in Korea (CISL-1504/ROSE study). Eligible participants were patients aged 20–65 years with previously untreated histologically confirmed PTCLs based on the World Health Organization classification 2008 including PTCL-NOS, AITL, ALK-negative ALCL, enteropathy-associated T-cell lymphoma (EATL), and hepatosplenic T-cell lymphoma (HSTL). Patients with ALK-positive ALCL, extranodal NK/T-cell lymphoma, and mycosis fungoides/Sezary syndrome were not included in the study. For participation, patients were required to have stage I–IV disease and an Eastern Cooperative Oncology Group performance status ≤ 2. Patients were not eligible if they did not have adequate cardiac, renal, hepatic, and bone marrow function. Thus, patients with an absolute neutrophil count < 1,500 cells/mm^3^ and platelet count < 100,000/mm^3^ (a platelet count < 75,000/mm^3^ in the case of bone marrow involvement) were excluded from the study. Patients with central nervous system or leptomeningeal involvement, positive serology for HIV-1, or active hepatitis B or C were also not eligible for the study. Consolidation treatment with ASCT was mandatory for all participants who responded to induction treatment. Patients were required to provide written informed consent before registration. The protocol was approved by the Institutional Review Boards of each participating institute, and the study was undertaken in accordance with the Declaration of Helsinki (ClinicalTrials.gov: NCT02445404).

### Procedures

After enrollment, patients underwent baseline assessments within 21 days before the first dose of the study drugs including blood tests, echocardiography, bone marrow aspiration/biopsy contrast-enhanced computed tomography (CT) scans of the neck, thorax, abdomen and pelvis, and Flourine-18 fluorodeoxyglucose positron emission tomography/computed tomography (¹⁸F-FDG-PET-CT) scans. Patients were assigned in a 1:1 ratio of CHOP to fractionated ICED by the CISL office according to two stratification variables: 1) histological subtypes (PTCL-NOS versus AITL) and 2) International Prognostic Index (IPI) risk (low/low-intermediate vs. high-intermediate/high). Treatment started within 4 days after randomization. Patients who were assigned to the CHOP group received cyclophosphamide 750 mg/m^2^, doxorubicin 50 mg/m^2^, and vincristine 1.4 mg/m^2^ (maximum 2 mg) intravenously on day 1, and oral prednisolone 100 mg on days 1–5 every three weeks, for six cycles. Patients in the ICED group received intravenous ifosfamide 1,670 mg/m^2^ on days 1–3, carboplatin 5 x area under the curve (AUC) on day 1, intravenous etoposide 100 mg/m^2^ on days 1–3, and oral or intravenous dexamethasone 40 mg on days 1–4 every three weeks, for six cycles. For both treatment groups, dose modifications were required for treatment-related toxicities in accordance with the study protocol. Pegylated granulocyte colony stimulating factor (G-CSF) was administered on day 2 after CHOP and day 4 after ICED. Administration of supportive medication, including antiemetic therapy was given in accordance with local practice. Prophylaxis for *Pneumocystis jirovecii* pneumonia was mandatory for all patients at each cycle whatever the treatment arm. The response evaluation was done by contrast-enhanced CT and ¹⁸F-FDG-PET-CT scans prior to the fourth cycle and after the sixth cycle of each treatment arm. Patients who achieved a complete or partial response after the completion of six cycles received ASCT as consolidation therapy within three months from the start of the sixth cycle of chemotherapy for both treatment arms. Peripheral blood stem cells for ASCT were collected during the fourth–sixth cycles of the treatment period or G-CSF mobilization after the completion of six cycles according to the investigator’s decision. The minimum CD34+ cell count was over 3.0 × 10^6^/kg of the recipient’s body weight. Intravenous administration of busulfan 3.2 mg/kg on day –7 to –5, etoposide 200 mg/m², twice a day on day –5 to –4, and cyclophosphamide 50 mg/kg on day –3 to –2 was done as conditioning chemotherapy prior to ASCT. After completion of the study treatment, patients were monitored by CT scan every three months for the first year, every four months for the next year, and every six months thereafter. If a patient showed any signs of clinically suspicious relapse or progression, investigators performed CT and ¹⁸F-FDG-PET-CT scans. Survival status was monitored after disease relapse or progression was confirmed.

### Outcomes

The primary end point was progression-free survival (PFS) at three years, and the PFS was defined from the date of the first day of the first cycle treatment until the date of the first observation of documented disease relapse or progression or any kind of death. Patients who received at least one cycle of treatment were defined as the assessable population, and the primary end point was assessed in the assessable population. Secondary end points were overall survival (OS) between the date of randomization and any kinds of death, an objective response rate (ORR) consisting of complete response (CR) and partial response (PR), and the safety outcomes of CHOP and ICED. Response evaluation and the assessment of stable disease (SD) or relapse/progressive disease (PD) were conducted by the participating investigators according to the Revised Response Criteria for Malignant Lymphoma (17). The interim and final response evaluation were performed after the third and sixth cycle of each treatment. Patients undergoing upfront ASCT after the completion of induction treatment were considered as the maintenance of response without any event for the estimation of PFS. Safety outcomes were graded according to the National Cancer Institute Common Terminology Criteria for Adverse Events (version 4.03). A subgroup analysis was done to assess factors associated with the primary end point based on subtypes of PTCLs and clinical and laboratory parameters. As an exploratory analysis, we compared the mutation profiles of tumor tissue at diagnosis with the treatment outcomes in patients who had archived targeted sequencing data available for analysis as previously described (18, 19). Mean sequencing coverage was greater than 700 x, and somatic alterations were called by a previously described pipeline: MuTect version 1.1.6, Lowfreq version 0.6.1, Pindel version 0.2.5a4 software, and a custom-built in-house algorithm (19–21).

### Statistical analyses

Sample size determination was based on the PFS. This study aimed to test the hypothesis that three-year PFS of ICED would be improved up to a rate of 60% compared with 40%, the rate expected for three-year PFS in the CHOP arm. Interim analysis was performed using group sequential design and, to correct the errors caused by multiple testing, the O’Brien–Fleming method was used. Interim analysis of PFS between the two groups was performed at 41 cumulative progression/death events with the two-sample log-rank test. If the standardized p-value of the log-rank test was greater than 0.686, the effectiveness of the ICED group was concluded as no better compared to CHOP, so the study was stopped. However, if the interim analysis result showed a p-value less than 0.017, it would be declared that the effectiveness of ICED therapy was statistically highly significantly better than CHOP, and the study would be terminated. If the p-value was between 0.017 and 0.686, the study would continue to proceed. The log-rank for one-sided alpha = 10% for 90% power required 134 patients (67 patients in each arm) and under the 2% drop-out assumption, a total 138 patients were enrolled. The final analysis was planned when at least 82 cumulative progression/death events occurred. Fisher’s exact test was used for associations between categorical variables. The Kaplan–Meier method was used to estimate OS and PFS, and the results were compared using the log-rank test. Data were analyzed using Statistical Package for Social Sciences software (version 24.0; IBM Corp, Armonk, NY, USA).

## Results

### Randomization and responses

**Figure 1:**
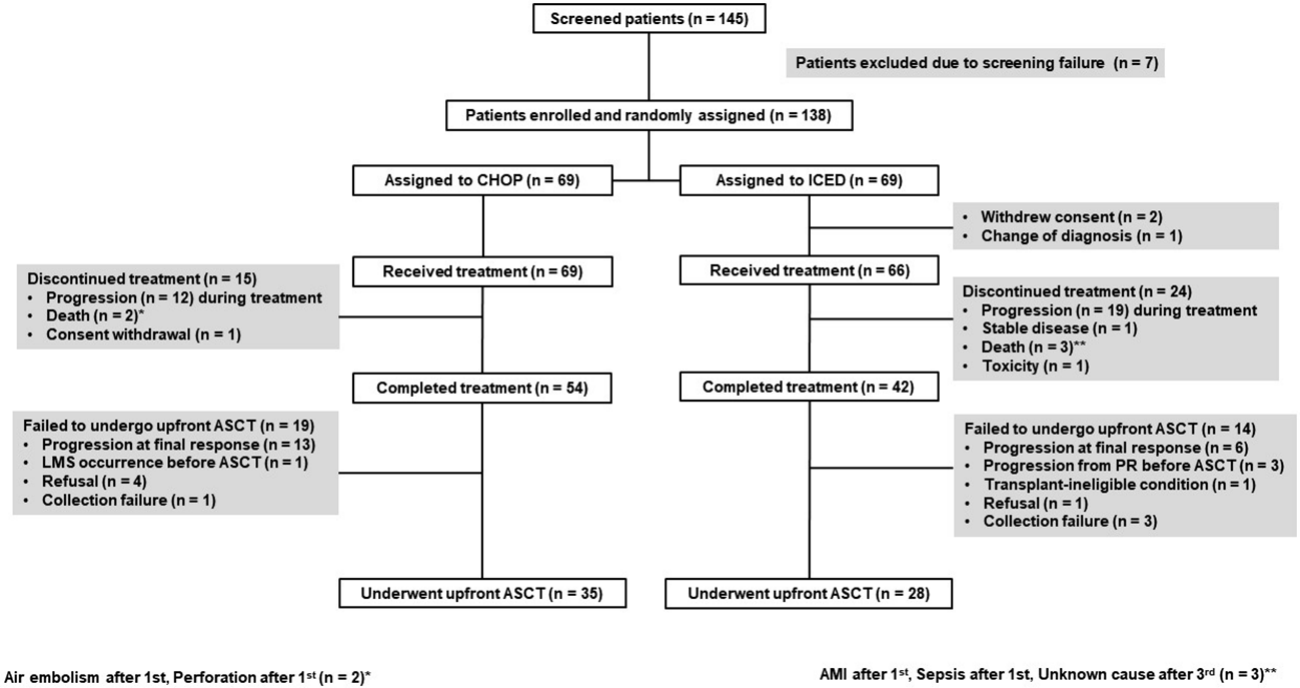
CONSORT diagram CHOP, cyclophosphamide, doxorubicin, vincristine, and prednisone; ICED, ifosfamide, carboplatin, etoposide, and dexamethasone. ASCT, autologous stem cell transplantation; LMS, leptomeningeal seeding; PR, partial response; AMI, acute myocardial infarction.

**Figure 2:**
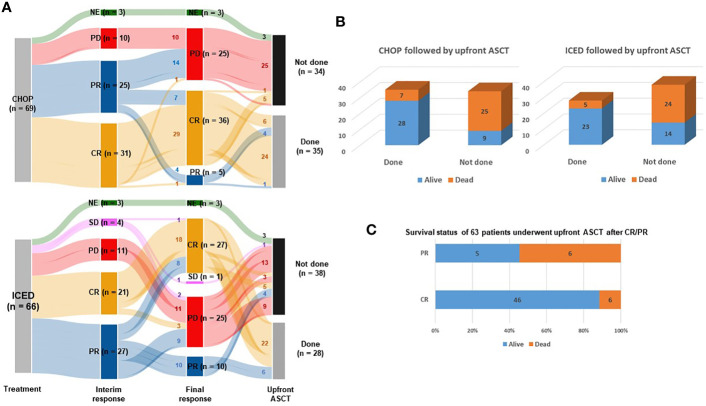
Response and outcomes of both arms. **(A)** Sankey diagram for treatment responses and conductance of upfront ASCT. CR, complete response; PR, partial response; SD, stable disease; PD, progressive disease; NF, not evaluated. Small letters in the lines represent the number of patients belonging to each line. **(B)** Comparison of surviving and non-surviving patients treated with CHOP and ICED followed by upfront ASCT. **(C)** Survival status of 63 patients who underwent upfront ASCT after CR/PR.

Between September 2015 and March 2021, 145 patients were screened, and 138 patients were enrolled (**Figure 1**). The interim analysis on PFS between the two groups in 2019 showed that the p-value for PFS difference was 0.640, and thus the study was continued until the final enrollment. After randomization, two patients withdrew informed consent, and one patient’s diagnosis was changed in the ICED group. Accordingly, 66 patients received the first cycle of ICED, whereas 69 patients received the first cycle of CHOP after assignment (**Figure 1**). The median age of 135 assessable patients was 57 years (range, 27–65 years), and the most common subtypes were PTCL-NOS (n = 61) and AITL (n = 53, **Table 1**). Although patients were randomized based on the IPI and subtype, a greater number of patients with ALK-negative ALCL and stage III/IV were assigned to the ICED group. However, there was no significant difference in baseline characteristics between the two arms (**Table 1**). In the CHOP arm, response evaluation was not done in three patients who dropped out after the first cycle: two deaths (one patient with EATL died due to bowel perforation, and the other patient died due to central catheter-related air embolism) and one patient refused to participate in the study. In the ICED arm, three patients’ responses were not evaluated because two patients died due to acute myocardial infarction and sepsis, respectively, after the first cycle, and the other patient died due to unknown causes after the third cycle (**Figure 1**). The interim response evaluation showed 31 CR, 25 PR, and 10 PD in the CHOP arm. Out of 25 patients with interim PR, only seven patients achieved CR, whereas 14 patients showed PD at the end of treatment. Finally, the ORR of CHOP was 59.4% (41/69), consisting of 36 CR and five PR (**Figure 2A**). The interim response to ICED showed 21 CR, 27 PR, four SD, and 11 PD. Out of 27 with interim PR, eight patients achieved CR, whereas nine patients progressed at the final response evaluation. Thus, the number of PR patients was 10 at final response, whereas 27 patients achieved CR at final response because three patients with interim CR progressed at final response. As a result, the ORR of ICED was 56.1% (37/66), consisting of 27 CR and 10 PR (**Figure 2A**). Although the number of patients with CR was different (CHOP: n = 36 vs. ICED: n = 27), the ORR was not different between CHOP (41/69, 59.4%) and ICED (37/66, 56.1%) arms (p = 0.421).

**Table 1:** Patient characteristics.

	Total (n = 135)	CHOP (n = 69)	ICED (n = 66)	P
Median age, years (range)	57 (27–65)	57 years (27–65)	57 years (29–64)	
Age Age ≤ 60 years Age > 60 years	99 (73.3) 36 (26.7)	50 (72.5) 19 (27.5)	49 (74.2) 17 (25.8)	0.848
Sex Male Female	85 (63.0) 50 (37.0)	47 (68.1) 22 (31.9)	38 (57.6) 28 (42.4)	0.218
Histologic subtype PTCL-NOS AITL ALK-negative ALCL EATL/HSTL	61 (45.2) 53 (39.3) 11 (8.1) 10 (7.4)	33 (47.8) 29 (42.0) 2 (2.9) 4/1 (7.2)	28 (42.4) 24 (36.4) 9 (13.6) 4/1 (7.6)	0.153
ECOG performance status 0 1 2	66 (48.9) 61 (45.2) 8 (5.9)	32 (46.4) 34 (49.3) 3 (4.3)	34 (51.5) 27 (40.9) 5 (7.6)	0.523
Ann Arbor stage I/II III/IV	20 (14.8) 115 (85.2)	2/12 (20.3) 32/23 (79.7)	1/5 (9.1) 24/36 (90.9)	0.067
B symptoms Absence Presence	81 (60.0) 54 (40.0)	45 (65.2) 24 (34.8)	36 (54.5) 30 (45.5)	0.223
Serum LDH Normal Increased	56 (41.5) 79 (58.5)	29 (42.0) 40 (58.0)	27 (40.9) 39 (59.1)	< 0.99
Extranodal involvement < 2 sites ≥ 2 sites	68 (50.4) 67 (49.6)	33 (47.8) 36 (52.2)	35 (53.0) 31 (47.0)	0.607
IPI risk Low/Low-intermediate High-intermediate/High	80 (59.2) 55 (40.8)	16/28 (63.8) 16/9 (36.2)	13/23 (54.5) 20/10 (45.5)	0.745
Bone marrow involvement Absence Presence	95 (70.4) 40 (29.6)	51 (73.9) 18 (26.1)	44 (66.7) 22 (33.3)	0.451
Serum albumin > 3.5 g/dL ≤ 3.5 g/dL	82 (60.7) 53 (39.3)	46 (66.7) 23 (33.3)	36 (54.5) 30 (45.5)	0.163
Absolute lymphocyte count > 1,000 mm^3^ ≤ 1,000 mm^3^	72 (53.3) 63 (46.7)	37 (53.6) 32 (46.4)	35 (53.0) 31 (47.0)	< 0.99
Hemoglobin ≥ 10 g/dL < 10 g/dL	106 (78.5) 29 (21.5)	57 (82.6) 12 (17.4)	49 (74.2) 17 (25.8)	0.296

PTCL-NOS, Peripheral T-cell lymphoma, not otherwise specified; AITL, Angioimmunoblastic T-cell lymphoma; ALCL, Anaplastic large cell lymphoma; EATL, Enteropathy-associated T-cell lymphoma; HSTL, Hepatosplenic T-cell lymphoma; ECOG, Eastern Cooperative Oncology Group; LDH, Lactate dehydrogenase; IPI, International Prognostic Index

The numbers in parentheses are percentages.

### ASCT and survival outcomes

Among 54 patients who completed six cycles of CHOP treatment, 13 patients with interim CR or PR showed PD after the completion of CHOP. Four patients with CR refused ASCT and stem cells were not collected from one patient. Another patient with CR failed to undergo ASCT because leptomeningeal involvement occurred before ASCT. Thus, 35 patients received upfront ASCT according to the protocol (**Figure 1**). In the ICED arm, 28 patients underwent upfront ASCT out of 42 patients who completed six cycles of ICED because of disease progression, collection failure, and refusal to undergo ASCT. Three patients with PR at the final response showed disease progression while they were preparing for ASCT (**Figure 1**). The number of patients who underwent the planned upfront ASCT was not significantly different between CHOP (35/69, 50.7%) and ICED (28/66, 42.4%) either (p = 0.389). The number of surviving patients was not different between the CHOP (37/69, 53.6%) and ICED groups (37/66, 56.1%, p = 0.863) at the time of analysis. The most common cause of death was disease relapse or progression in both arms, and most deaths occurred during the post-treatment period. The survival outcome was significantly better in patients who completed the treatment protocol including upfront ASCT in both arms (**Figure 2B**). When the survival status of 63 patients who underwent upfront ASCT was compared according to the final response to CHOP or ICED treatment, patients with PR showed poor survival (**Figure 2C**).

### Analysis for PFS

**Figure 3:**
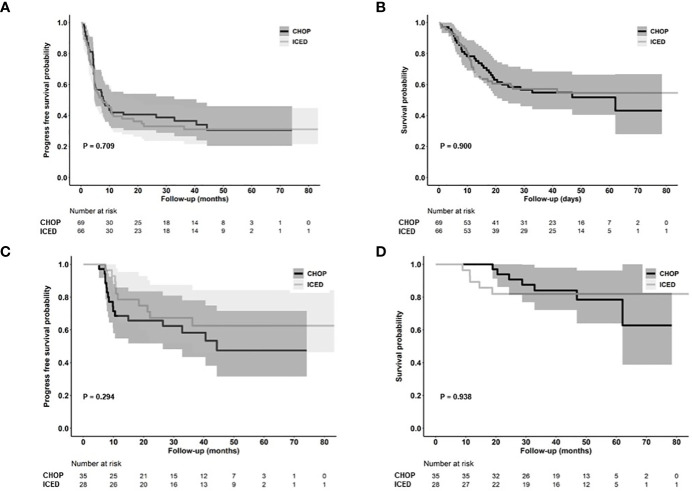
Survival outcomes. **(A, B)** Kaplan–Meier estimates of PFS and OS in the ICED and CHOP groups. **(C, D)** Kaplan–Meier estimates of PFS and OS in patients who completed the treatment protocol including upfront ASCT of ICED and CHOP group. CHOP, cyclophosphamide, doxorubicin, vincristine, and prednisone; ICED, ifosfamide, carboplatin, etoposide, and dexamethasone; PFS, progression-free survival; OS, overall survival.

**Figure 4:**
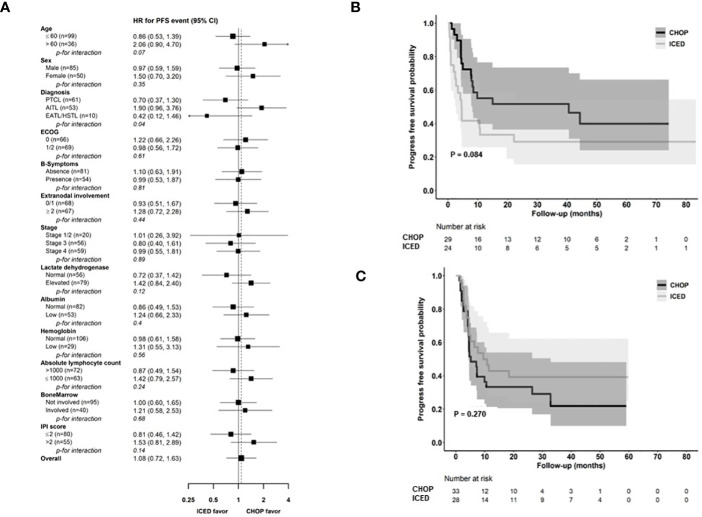
Comparison of PFS based on parameters **(A)** Subgroup analysis for PFS. **(B, C)** Kaplan–Meier estimates of PFS in patients with AITL and PTCL-NOS. CHOP, cyclophosphamide, doxorubicin, vincristine, and prednisone; ICED, ifosfamide, carboplatin, etoposide, and dexamethasone; PFS, progression-free survival; PTCL-NOS, Peripheral T-cell lymphoma, not otherwise specified; AITL, Angioimmunoblastic T-cell lymphoma; EATL, Enteropathy-associated T-cell lymphoma; HSTL, Hepatosplenic T-cell lymphoma; ECOG, Eastern Cooperative Oncology Group; IPI, International Prognostic Index. * ALK-negative Anaplastic large cell lymphoma was excluded because of the small number of patients in the CHOP group.

**Figure 5:**
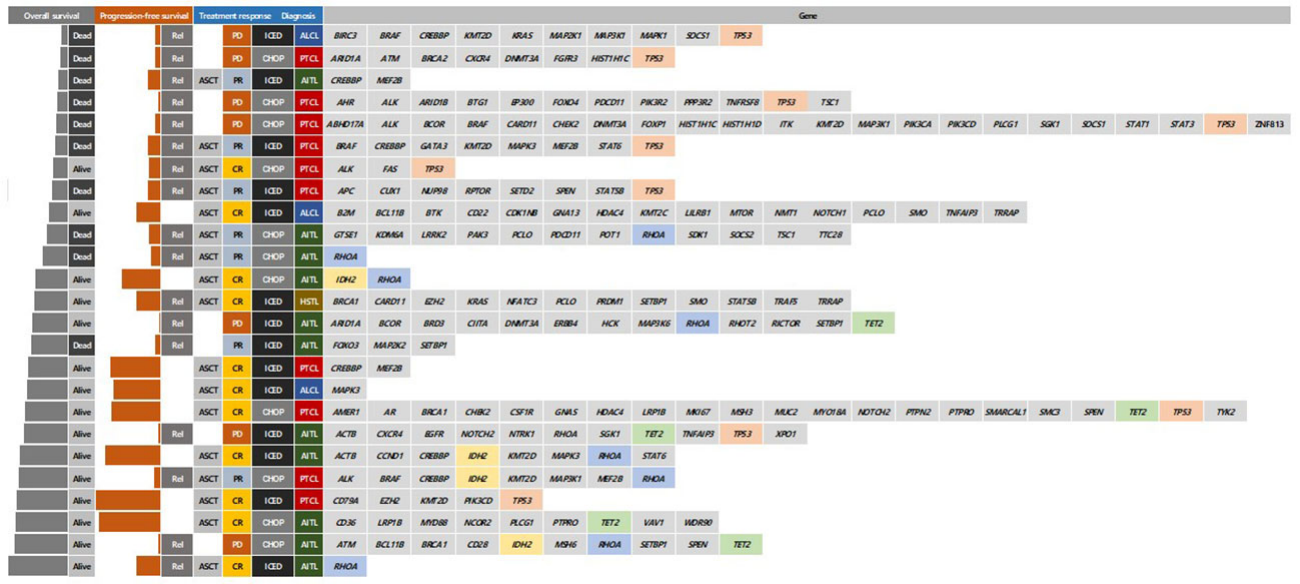
Comparison of survivals and responses according to mutation profiles at diagnosis Rel, relapse; ASCT, autologous stem cell transplantation; CR, complete response; PR, partial response; PD, progressive disease; CHOP, cyclophosphamide, doxorubicin, vincristine, and prednisone; ICED, ifosfamide, carboplatin, etoposide, and dexamethasone; PFS, progression-free survival; PTCL-NOS, Peripheral T-cell lymphoma, not otherwise specified; AITL, Angioimmunoblastic T-cell lymphoma; HSTL, Hepatosplenic T-cell lymphoma.

As of 31 December 2022, after a median follow-up of 44.8 months (95% Confidence Interval [CI]: 40.8–48.8 months), the occurrence of relapse or progression was not significantly different between the CHOP (28/69, 40.6%) and ICED groups (24/66, 36.4%, p = 0.724). Accordingly, the primary end point of PFS was not met, and the three-year PFS was not significantly different between the CHOP (36.7%) and ICED groups (33.1%, p = 0.709, **Figure 3A**). Among the patients who had relapse or progression after CHOP or ICED, seven patients in the ICED arm underwent salvage ASCT after they responded to subsequent therapy, whereas three patients in the CHOP arm underwent salvage ASCT. Thus, the three-year OS was not significantly different between the CHOP (54.9%) and ICED groups (57.2%, p = 0.900, **Figure 3B**). Patients who completed the CHOP or ICED treatment followed by upfront ASCT according to the protocol did not show a significant difference in PFS (p = 0.294, **Figure 3C**) and OS (p = 0.938, **Figure 3D**) either. The PFS analyses were conducted in subgroups with potential prognostic factors for 135 patients. There was no statistically significant difference in PFS between the CHOP and ICED arms for any subgroup analyzed except subtypes such as AITL (**Figure 4A**). Thus, CHOP was favored over ICED in AITL patients, whereas ICED was favored over CHOP in EATL/HSTL patients (**Figure 4A**). Accordingly, the comparison showed a better PFS of AITL in CHOP than ICED with a marginal significance (**Figure 4B**). On the other hand, ICED showed a trend toward better PFS in PTCL-NOS patients, although it was not statistically significant (**Figure 4C**). Exploratory analysis of PFS in a group of patients (n = 25) whose sequencing data were available showed worse survival outcomes in patients with TP53 mutations regardless of treatment, although it was not statistically significant (**Figure 5**).

### Adverse events

Treatment-emergent adverse events were assessed in 135 patients who received at least one dose of the study treatment. CHOP was associated with more peripheral neuropathy of all grades (**Table 2**). ICED was associated with more anemia, neutropenia, and thrombocytopenia of all grades (**Table 2**). Accordingly, the frequency of febrile neutropenia was higher in the ICED arm vs. the CHOP arm although it was not statistically significant (19.7% vs. 10.1%, p = 0.143). Grade 4 adverse events were higher in the ICED (n = 43) arm vs. CHOP (n = 8) arm (65.1% vs. 11.6%, p < 0.001). As a result, the relative dose intensity (calculated as the average of the four) drugs for ICED (92.5%) was lower than that for CHOP (98.8%) because of the dose reduction and delay in the ICED arm.

**Table 2:** Adverse events by treatment.

	CHOP (n = 69)			ICED (n = 66)		
	All grades (%)	G3 (%)	G4 (%)	All grades (%)	G3 (%)	G4 (%)
Acute appendicitis	0	1 (1.4)	0	0	0	0
Anal abscess	0	0	0	0	1 (1.5)	0
Anemia	7 (10.1)	3 (4.3)	0	23 (33.3)	16 (24.2)	2 (3.0)
Anorexia	7 (10.1)	0	0	11 (16.7)	0	0
Constipation	8 (11.6)	0	0	13 (19.7)	0	0
Diarrhea	5 (7.2)	2 (2.9)	0	1 (1.5)	1 (1.5)	0
Fatigue	10 (14.5)	0	0	11 (16.7)	3 (4.5)	0
Febrile neutropenia	7 (10.1)	7 (10.1)	0	13 (19.7)	11 (16.7)	2 (3.0)
Fever	11 (15.9)	1 (1.4)	0	12 (18.1)	0	0
Insomnia	1 (1.4)	0	0	8 (12.1)	0	0
Mucositis	12 (17.4)	1 (1.4)	0	7 (10.6)	1 (1.5)	0
Nausea	10 (14.5)	0	0	28 (42.4)	0	0
Neutropenia	11 (15.9)	4 (5.8)	7 (10.1)	23 (34.8)	4 (6.1)	18 (27.3)
Peripheral neuropathy	16 (23.2)	0	0	3 (4.5)	0	0
Pneumonia	4 (5.8)	2 (2.9)	0	3 (4.5)	3 (4.5)	0
Respiratory failure	0	0	0	1 (1.5)	0	1 (1.5)
Sepsis	1 (1.4)	0	1 (1.4)	2 (3.0)	0	2 (3.0)
Skin rash	4 (5.8)	0	0	12 (18.1)	2 (3.0	0
Thrombocytopenia	12 (17.4)	8 (11.6)	0	29 (43.9)	5 (7.6)	18 (27.3)
Vomiting	2 (2.9)	0	0	6 (9.1)	0	0
Other*	3 (4.3)	3 (4.3)	0	4 (6.1)	4 (6.1)	0

*CHOP: enterocolitis grade 3 (n = 1); cellulitis grade 3 (n = 1); arrhythmia grade 3 (n = 1); two deaths occurred after the first cycle (one patient with EATL died due to bowel perforation, and the other patient died due to central catheter-related air embolism). *ICED: syncope grade 3 (n = 1); urinary tract infection grade 3 (n = 2); vancomycin-resistant enterococcal infection grade 3 (n = 1); two deaths occurred after the first cycle (acute myocardial infarction and sepsis, respectively), and one death with unknown cause occurred after the third cycle.

## Discussion

This study is the first randomized trial in a first-line setting comparing ifosfamide containing a non-anthracycline intensified regimen, ICED, with CHOP across various PTCLs. We designed a randomized phase II study that could be more feasible than phase III because the relatively low incidence of PTCLs could influence the accrual rate and sample size (22). Our hypothesis was that the intensified non-anthracycline regimen of ICED followed by ASCT might be more effective than CHOP in terms of PFS because the efficacy of the ICE regimen was already proven for relapsed or refractory lymphoma and ICED followed by ASCT showed more than 40% of PFS in 75 patients with relapsed lymphoma (15). However, the final number of patients with CR was lower in ICED (n = 27) than in CHOP treatment (n = 36, **Figure 2A**). Thus, the efficacy of ICED in terms of CR rate in a first-line setting was inferior to CHOP, although the ORR was not different between the CHOP (41/69, 59.4%) and ICED (37/66, 56.1%) arms (p = 0.421). The number of patients with PD was also the same between the two arms (n = 25). Out of 25 interim PR patients, 14 patients showed PD (56.0%, 14/25) in the CHOP arm, while nine interim PR patients in the ICED arm finally showed PD (33.3%, 9/27, **Figure 2A**). Accordingly, the number of patients who underwent upfront ASCT was not significantly different between ICED (42.4%, 28/66) and CHOP (50.7%, 35/69, p = 0.389) treatments. The three-year PFS was not significantly different between CHOP and ICED therapies either (**Figure 3A**), and the three-year PFS of both arms (CHOP, 36.7% and ICED, 33.1%) was lower than the expected 40% three-year PFS of our study design. Thus, our study failed to demonstrate the superiority of ICED over CHOP, and the three-year OS was not improved with ICED treatment compared with CHOP treatment (57.2% and 54.9%, respectively, **Figure 3B**). While patients who completed the CHOP or ICED program followed by upfront ASCT showed around 80% three-year OS (**Figure 3D**), more than a half of patients receiving ASCT at PR died due to disease relapse (**Figure 2C**). In addition, the subgroup analysis for PFS with potential prognostic factors showed that CHOP was favored over ICED in AITL patients (**Figures 4A, B**). On the other hand, ICED showed a trend toward better PFS in PTCL-NOS patients, although this was not statistically significant (**Figure 4C**), and ICED was favored over CHOP in EATL/HSTL patients, although the number of patients was small (**Figure 4A**). Safety profiles showed that ICED was associated with more cytopenia of grade 3 or worse (**Table 2**), resulting in lower relative dose intensity than CHOP due to dose reduction and delay in the ICED arm. These hematologic toxicities related to the intensified dosing regimen were associated with more frequent occurrences of febrile neutropenia as well as stem cell collection failure (**Table 2**).

Taken together, our data show that ICED is inferior to CHOP for treatment-naive patients with PTCLs because it fails to demonstrate superiority in terms of PFS and safety profiles. Nevertheless, our results suggest several directions for the future management of newly diagnosed, treatment-naive patients with PTCLs. First, our study started before the results of large study population-based randomized studies were reported. Thus, we did not incorporate novel drugs into the experimental arm and only compared the outcomes of conventional cytotoxic chemotherapies. Even so, the RO-CHOP study failed to demonstrate the benefit of romidepsin plus CHOP compared to CHOP alone because of increased treatment-related toxicities without significant improvement in survival (23). Likewise, the ACT-2 trial also failed to show the superiority of alemtuzumab plus CHOP over CHOP in elderly patients with PTCLs because of treatment-related toxicity, even though alemtuzumab-CHOP increased response rates (24). Based on the favorable outcome of CHOEP in the previous German study group (11), the PTCL13 phase Ib/II study analyzed the efficacy of romidepsin plus CHOEP followed by upfront ASCT in untreated patients with PTCLs (25). However, they reported 46.2% of the 18-months PFS and failed to meet the primary endpoint.

The ECHELON-2 trial demonstrated a superior outcome with brentuximab vedotin plus cyclophosphamide, doxorubicin, and prednisone (CHP) for patients with CD30-positive PTCLs. However, CD30 was not expressed in all patients with PTCLs and the overall benefit of this regimen could be expected mainly in ALCL (26). Like our study, a previous randomized phase II study compared CHOP with a non-anthracycline regimen, gemcitabine, cisplatin, and methylprednisolone (GEM-P). However, the study closed early to recruitment because GEM-P was non-significantly inferior to CHOP (27). Another phase II randomized trial comparing cyclophosphamide, epirubicin, vincristine, and prednisone (CEOP), ifosfamide, epirubicin, and etoposide (IVE), and gemcitabine, cisplatin, and dexamethasone (GDP) in an alternating regimen with CEOP in newly diagnosed PTCL showed no remission or survival advantage to standard chemotherapy, similar to our study (28). These results imply that there are still unmet needs for the treatment of newly diagnosed, treatment-naive patients with PTCLs. Importantly, our results showed that CHOP could induce a favorable outcome in patients with AITL, although the efficacy of CHOP was unsatisfactory in patients with PTCL-NOS and EATL/HSTL. These findings imply that clinical trials should be designed according to subtype of PTCLs because one size does not fit all. Thus, CHOP could be used as the backbone for a new combination treatment for newly diagnosed follicular T-helper cell-derived lymphomas including AITL. Indeed, our group is currently performing a phase II trial of azacitidine-CHOP for patients with previously untreated T-follicular helper phenotype (ClinicalTrials.gov: NCT05230680). Likewise, the efficacy of intensified non-anthracycline based regimens should be explored for patients with PTCL-NOS and EATL/HSTL in future studies given their favorable outcomes in our study.

Second, our study prospectively demonstrated the clinical relevance of PR determined by PET/CT scans. Most patients with interim PR showed disease progression at the final response evaluation, which is consistent with a previous study reporting that the interim PET response could stratify risk of treatment failure in patients with PTCLs (29). Furthermore, patients receiving upfront ASCT at CR showed better outcomes than patients with PR prior to upfront ASCT in our study (**Figures 2B, C**). Patients receiving ICED or CHOP followed by upfront ASCT showed a plateau in the survival curve and around 80% of three-year PFS (**Figure 3D**). This outcome was better than the 44% of five-year event-free survival in the NLG-T-01 study (14). Although the role of upfront ASCT is still not clear in newly diagnosed PTCLs, a recent population-based cohort study showed the impact of ASCT on survival among patients aged < 65 years with stage II to IV PTCL (30). Similarly, a recent exploratory subgroup analysis for patients enrolled in the ECHELON-2 study demonstrated the role of consolidative ASCT in patients with CD30-positive PTCL who achieved CR following treatment with brentuximab vedotin plus CHP (31). Therefore, the development of regimens achieving CR and active application of consolidative ASCT could improve the ultimate outcome of patients with PTCLs. Our exploratory analysis with the sequencing data showed worse survival outcome of patients with TP53 mutations regardless of treatment. Thus, the addition of novel drugs overcoming the unfavorable biological characteristics should be considered for the achievement of CR.

In conclusion, our phase II randomized study showed no difference between CHOP and ICED, and the outcome of both treatments was comparable to that of previous studies. Considering the different outcomes according to subtypes of PTCLs, CHOP should remain the reference regimen for AITL, whereas other regimens should be investigated for PTCL-NOS and EATL/HSTL. The favorable outcome of patients receiving upfront ASCT at CR after ICED or CHOP implies a role for ASCT as a consolidation of CR state regardless of the type of induction regimens, although further studies are warranted.

## Data availability statement

The original contributions presented in the study are included in the article/supplementary materials, further inquiries can be directed to the corresponding author/s.

## Ethics statement

This study was approved by the Institutional Review Boards of the participating institutes. It was conducted in accordance with the ethical principles of the Declaration of Helsinki and the Korea Good Clinical Practice guidelines.

## Author contributions

SK, J-CJ, and WK designed the research and wrote the primary manuscript. SK, J-CJ, WK, DoHY, DeHY, SY, G-WL, JK, YP, K-WK, H-SL, SO, H-JS, HK, WL, YC, SJ, MK, JY, S-NL, HYY, YD, HJY, H-SE, ML, and CS performed the research and collected the patients’ data. All authors edited the manuscript and approved the final version.
